# Discrimination between bacterial species by ratiometric analysis of their carbohydrate binding profile†
†Electronic supplementary information (ESI) available: Experimental procedures. See DOI: 10.1039/c5mb00720h



**DOI:** 10.1039/c5mb00720h

**Published:** 2015-12-09

**Authors:** Lucienne Otten, Elizabeth Fullam, Matthew I. Gibson

**Affiliations:** a Department of Chemistry , University of Warwick , Gibbet Hill Road , Coventry , CV4 7AL , UK . Email: m.i.gibson@warwick.ac.uk; b School of Life Sciences , University of Warwick , Gibbet Hill Road , Coventry , UK

## Abstract

Antibiotic resistance is a global health concern meaning there is an urgent need for new treatments and diagnostics. Here glycosylated surfaces are used to profile the binding patterns of a range of Gram-negative, Gram-positive and mycobacteria. This enables the creation of ‘barcodes’ to enable identification and discrimination between the strains, which could not be achieved by single-point glycan binding and offers a new concept in bacteria detection.

## Abstract 2

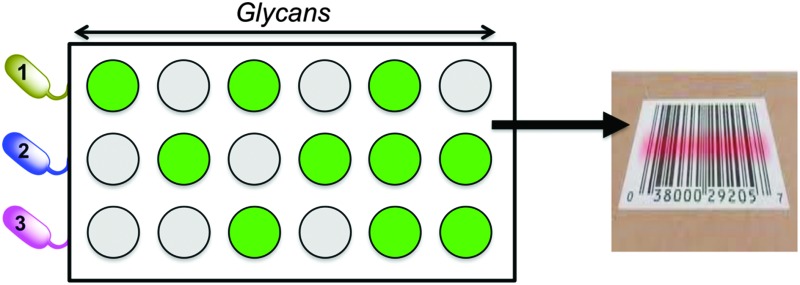
‘Barcoding’ how bacteria bind to simple sugar-surfaces using a training algorithm is used as a powerful identification tool.

Bacteria cause diseases including meningitis, pneumonia, diarrhea and many hospital acquired infections such as *Staphylococcus aureus* ^1^ or *Clostridium difficile* ^2^ and are responsible for millions of deaths every year. ‘Old’ diseases such as tuberculosis, caused by the bacterium *Mycobacterium tuberculosis*, for which antibiotics had previous proven to be effective, are once again on the rise^3–5^ with an estimated 2 billion carriers globally. Since penicillin was discovered in 1929 it revolutionized the treatment of bacterial infections^6^ but the current drug pipeline has dried up alongside development of resistance.^7^ The spread of resistance is very rapid; for example methicillin was clinically used in 1960 with the first case of methicillin resistant *S. aureus* reported in 1961.^8^ These factors have contributed to the prediction that by 2050 the number of deaths associated with antimicrobial resistance will outnumber those caused by cancer.^9,10^ The problem of antibiotic resistance is propagated by the lack of rapid point-of-care diagnostics tools to ensure correct use of the remaining drugs. Most of the current Food and Drug Administration (FDA) approved detection methods rely on a culturing step; the bacteria are isolated and grown for visual, or other examination methods, which can take several days^11^ and some strains are intrinsically challenging to culture. Since Robert Koch first described culturing for identification of bacteria over 140 years ago the techniques used has hardly changed since.^12,13^ However biofilm based infections are now becoming the most common problem, with these bacteria proving incredibly challenging to culture.^14^ It is estimated that only 1% of bacteria are culturable on standard culture media and several of the species that commonly cause hospital acquired infections can enter a viable but not culturable phase where they are not detectable through culturing techniques.^12,14,15^ The emerging alternative is the use of polymerase chain reaction (PCR) or sequencing methods. PCR based processes include; (i) the analysis of the full genome of microbial species and identification through comparison with a database^16,17^ and (ii) Ibis, a method which involves selective PCR of a species-specific element of DNA which is flanked by highly conserved regions. Whilst PCR based methods are very rapid (with Ibis reportedly only taking 6 hours) they are still relatively expensive (Ibis needs a mass spectrometer) and rely on extraction of nucleic acids from within bacteria, which can be problematic in Gram-positive species due to their thick peptidoglycan wall, which is difficult to lyse.^14^

Many bacteria infections require an initial adhesion step, for example, the high mannose-binding adhesin FimH is a crucial virulence factor found in uropathogenic *Escherichia coli* and among enterobacteriacae.^18,19^ Biofilm based infections are responsible for many chronic bacteria associated conditions such as pneumonia^20^ and tuberculosis^21^ and also the most difficult to culture.^22–24^ Considering the above, adhesins are potential targets for novel prophylactic anti-adhesion therapies^25,26^ or as diagnostics.^27–30^ Several nanoparticle-based detections systems exploiting adhesins have been reported including galabiose particles for the detection of *Streptococcus suis*.^20,31^ Mannose conjugated gold nanoparticles have been shown to detect FimH positive strains of *E. coli* in a colorimetric assay.^27^ These particle-based systems highlight the potential for carbohydrates in detection but are intrinsically limited by the promiscuity of glycan–lectin (carbohydrate binding proteins) interactions. Therefore, specific identification of lectins (or bacteria) is complicated by the non-specific binding which can give false positives. To overcome this, we have reported the use of a powerful statistical analysis technique for the identification of carbohydrate binding toxins with similar binding specificity using linear discriminant analysis (LDA) – essentially we created a ‘bar code’ to described each lectin.^32^ Here we demonstrate a multiplexed bacteria assay based on their relative binding affinity to a small and accessible array of glycans. The relative binding affinity of each bacteria was determined to generate a training matrix, which can subsequently be used for discrimination/identification. A range of Gram-negative, Gram-positive including mycobacteria are studied, including surrogates for *M. tuberculosis.*

The key aim here was to assess if the relative binding profiles of different bacterial strains to a range of glycans could be used to generate a training matrix as an easy method to enable multiplexed diagnostics based on each strains relative binding to different sugars. To generate glycosylated surfaces we employed hydrazide functional 96 well plates (Carbo-BIND™). Glycosylation with reducing carbohydrates was enabled by simply heating at 50 °C with an aniline catalyst. This method gives a mixture between ring-closed (approximately 60%) and ring opened sugars. Whilst generating some heterogeneity, the discrimination method we employ (see later) is not affected by this, and in theory any other glycan immobilisation method could be used. To enable visualisation of bacterial binding for this proof-of-concept study, a two-step labelling procedure was employed. Bacteria were first biotinylated with Biotin-NHS, followed by reaction with FITC-labelled streptavidin (Fig. 1A). We choose this method to enable facile binding measurement rather than as a true diagnostic tool. Alternative detection methods include microscopy, SPR, bilayer interferometry or nanoparticle binding, but are outside the scope of this manuscript. Successful labelling of the bacteria was showed by measuring the fluorescence (Fig. 1B) of the bacterial cultures and visual examination.

**Fig. 1:**
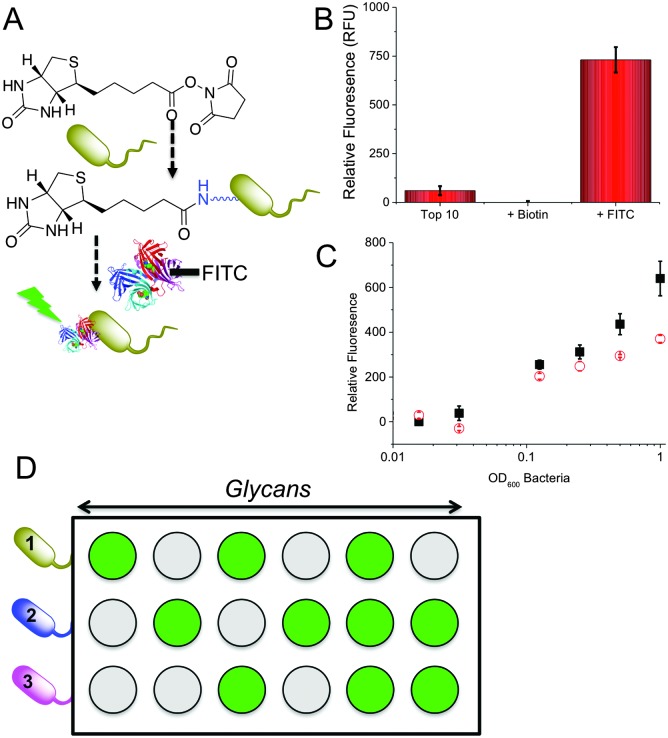
General concept employed here. (A) Fluorescent labelling of bacteria; (B) demonstrating of dye conjugation; (C) binding to glucosylated surface of labelled Top10 (red circle) and K12 (black square) *E. coli*; (D) discriminatory approach used here, where each bacteria will generate a different binding pattern.

To evaluate glycan binding, Top10 and K12 (*E. coli*) were incubated with a glucose functional surface as a function of bacterial concentration. A clear dose-dependant response was observed with the K12's showing more adherence at higher ODs (defined as the absorbance at 600 nm), which we attribute to their expression of FimH (Fig. 1C). This experiment also serves to highlight the challenge of single-channel glycan-based sensors – both bacteria would give a positive response. Fig. 1D shows the multiplexed concept to be used here. Looking ‘across’ from any given glycan, it would not be possible to identify the bacteria. However, using multiple glycan ‘inputs’ enables a barcode to be created which is unique to the bacteria. It is therefore clear that several glycans are required to gain sufficient resolution. 9 different glycans were immobilised onto the plates and 5 different bacteria strains (labelled as described above) were interrogated. The bacterial species examined here are; two strains of *E. coli* Top10 and K12, *Pseudomonas putida*, *Mycobacterium smegmatis* and *Mycobacterium marinum*. Of the two strains of *E. coli*: Top10 is FimH negative and K12 is FimH positive and thus a mimic of pathogenic strains. *P. putida* is similar in nature to *Pseudomonas aeruginosa* which causes life threatening infections that can affect multiple organs but commonly causes pneumonia in cystic fibrosis patients.^33,34^ *M. marinum* and *M. smegmatis* are both model organisms for *M. tuberculosis*, the causative agent of tuberculosis, with *M. marinum* sharing around 3000 orthologs with over 85% amino acid identity with *M. tuberculosis*.^35^ The species analysed included Gram-positive and Gram-negative species, pathogenic and non-pathogenic samples and different strains of the same species providing a focussed, but diverse set of bacteria. The relative binding of these bacteria to each surface was evaluated by fluorescence and the profiles are show in Fig. 2A.

**Fig. 2:**
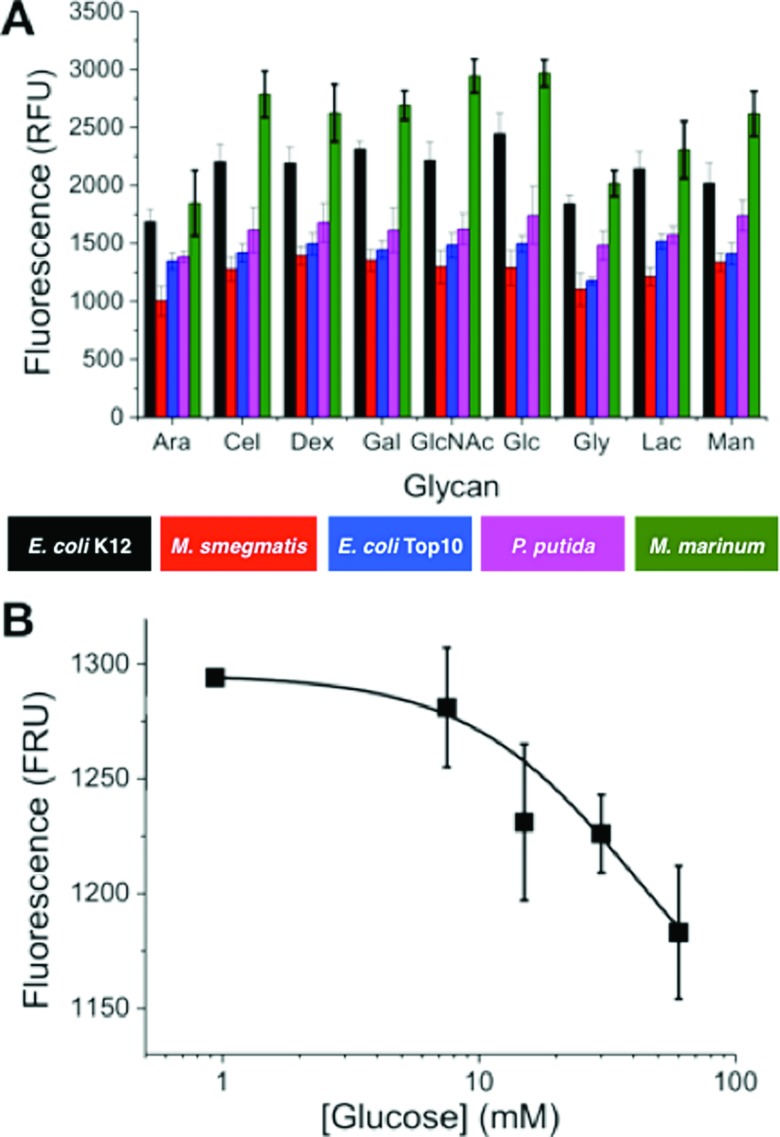
(A) Binding profiles of fluorescently labelled bacteria to glycan surfaces. Arabinose (Ara), cellobiose (Cel), dextran (Dex), galactose (Gal), glucose (Glc), *N*-acetyl-d-glucosamine (GlcNAc), glyceraldehyde (Gly), lactose (Lac) and mannose (Man). Each bar represents the average of 8 replicates and the error bars represent the standard error; (B) competitive binding of *M. smegmatis* to glucose surface, in presence of free glucose. Error is SD from minimum of 3 repeats.

Fig. 2A shows that individual strains exhibited distinct levels of adhesion. For example *M. marinum* showed higher binding to most glycans than the others. It is beyond the scope of this study to identify the individual components responsible for the binding, and it should be highlighted that this knowledge is not essential for this biosensory approach (*vide infra*). However, it was important to confirm the interactions were due to glycan–adhesion, and not just non-specific binding. A competition experiment was conducted whereby OD = 1 *M. smegmatis* was incubated with the surfaces, but with increasing glucose concentrations in the buffer (1–50 mM). As the glucose concentration was increased, there was a clear decrease in the amount of binding (Fig. 2B).

There are many putative carbohydrate binding proteins (including transporters) in the strains, and any given adhesin can also bind to other glycans, even if it has relatively lower affinity. Considering this, any single sugar cannot identify a specific bacteria, within the error of the measurement. However, each bacteria has a distinct set (‘barcode’) of binding across the range of sugars. This large set of data was thus used as a training matrix for LDA.^32,36^ LDA is a powerful classification tool, which for every class (bacterial species) within the training matrix (binding profiles) it minimises the variation within each class and maximises the variation between classes by transforming the original data in order to get maximum separation between groups. For the bacterial binding data, the LDA model produced showed good resolution between bacterial species and when validated using a ‘leave-one-out’ approach (where each sample is left out in an iterative process and the model generated before classification of the missing sample) the model was able to re-classify all data points to their original class with 92% accuracy. Fig. 3 shows a 2-dimensional representation of the LDA analysis and confidence boundaries for each bacterial strain. This clearly shows that each bacteria has a distinct ‘region’ enabling discrimination. In particular, the two mycobacterial species (*M. smegmatis* and *M. marinum*) had excellent separation.

**Fig. 3:**
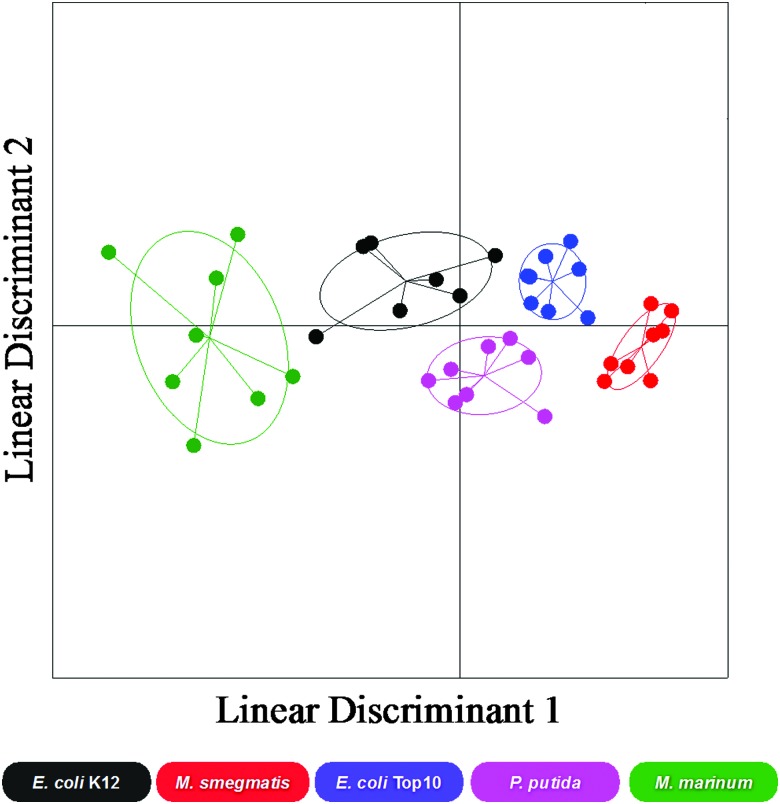
Linear discriminant analysis model of bacterial binding. *E. coli* K12, *E. coli* Top10, *M. marinum*, *P. putida* and *M. smegmatis*. Each point represents a single binding profile to all the glycan surfaces, the centre point dictates the average value for all samples and the ellipses represent one standard deviation from the average.

Finally a blind test was done. A culture was prepared by an independent operator and provided. This was fluorescently labelled and incubated with each of the glycan surfaces in triplicate. The average binding response was classified using the LDA model and was correctly identified as *E. coli* Top10 with 96% certainty. Future work will involve the extension of this method to ‘label-free’ analytical techniques and also focussing on clinical strains. The rapid and simple nature of this makes it ideal for translation and compares favourable with sequencing/PCR based methods such as Ibis which takes at least 6 hours for identification,^14^ and in some cases expansion and culture of the bacteria.

To conclude, we have demonstrated a rapid technique for the identification of bacterial strains based on profiling their differential binding to carbohydrate functionalised surfaces coupled with a powerful, but simple to use, classification algorithm. The procedure was shown to be valid for Gram-negative and Gram-positive/mycobacteria, indicating it has potential to diagnose or identify a broad range of pathogenic bacteria, either on its own, or in combination with other established analytical methods. In particular, the potential for rapid and low-cost diagnostics is shown including for mycobacteria. *M. tuberculosis* is a re-emerging global healthcare threat with no suitable detection methods.

We would like to thank Dr Alasdair Hubbard for technical assistance. Equipment was supported by the Birmingham Science City (SC) Advanced Materials project, with support from Advantage West Midlands and part funded by the European Regional Development Fund. LO is funded by the BBSRC *via* the Systems Biology DTC. EF is a Sir Henry Dale Fellowship jointly funded by the Wellcome Trust and the Royal Society (Grant Number 104193/Z/14/Z). MIG thanks the ERC for a Starting Grant, CRYOMAT 638661.
