# Supplementary material for: Discrimination between bacterial species by ratiometric analysis of their carbohydrate binding profile
Source: Mol Biosyst. 2015 Dec 9;12(2):341–4. doi: 10.1039/c5mb00720h (PMC4743676; doi:10.1039/c5mb00720h)
Supplement: Supplementary file 1 [file MB-012-C5MB00720H-s001.pdf]

Electronic Supplementary Material (ESI) for Molecular BioSystems.
This journal is © The Royal Society of Chemistry 2015

Supporting Information for

Discrimination between Bacterial Species by Ratiometric analysis of their Carbohydrate Binding Profile

Lucienne Otten[a], Elizabeth Fullam[b] and Matthew I. Gibson[a,*]

[a] Department of Chemistry, University of Warwick, Gibbet Hill Road, Coventry UK, CV4 7AL.

[b] School of Life Sciences, University of Warwick, Gibbet Hill Road, Coventry, UK, CV4 7AL.

[*] Corresponding Author email; m.i.gibson@warwick.ac.uk

Materials

All chemicals were used as supplied unless otherwise stated. Corning® 96 well clear flat bottomed polystyrene Carbo-BIND™ microplates, ExtrAvidin®-Fluoroscein isothiocyanate, (+)-Biotin N-hydroxysuccinimide ester, aniline (99.5 % ACS reagent), sodium acetate anhydrous, acetic acid glacial, phosphate buffered saline tablets, D-(+)galactose, DL-glyceraldehyde, lactose, L-arabinose, D-(+)cellobiose, dextran from *Leuconostoc mesenteroides*, D-(+)mannose, N-Acetyl-D-glucosamine, α-D-glucose and DMSO were all purchased from Sigma-Aldrich. 100 mM acetate buffer (pH 5.5) with 1 mM aniline was prepared in 200 mL of milliQ water (with a resistance of >19 mOhms). *E. coli* strains K12 JM109 (referred to as K12 in the text) and Top10 and *Pseudomonas putida* were grown in LB media. *Mycobacterium smegmatis mc^{2}155* was grown in LB broth supplemented with 0.05% Tween 80 and *Mycobacterium marinum M* was grown in 7H9 media (Difco). All strains were grown to late log phase in the indicated media and then used immediately.

Methods

Carbohydrate surface preparation

100 µL of 30 mM sugar solution (in 100 mM acetate buffer with 1 mM aniline, pH 5.5) was added to each well of a Carbo-BIND 96-well plate. Plates were then covered in foil and incubated at 50 °C for 24 hours. After incubation, any unbound solution was removed and the well washed with PBS. Plates were either used immediately or stored at -20 °C prior to their use.

Biotinylation of bacteria

Bacteria in cell culture media were centrifuged at 8000 rpm for 10 minutes and then the pellets were resuspended in PBS with a final OD of 1. Then 2.5 μL of (+)-Biotin N-hydroxysuccinimide ester (10 mg/mL in DMSO) was added to every 1 mL of bacterial solution before incubation at room temperature for 2.5 hours. A 200 μL sample was then removed and absorbance at 280 nm measured using a BioTek Synergy HT multi-detection microplate reader to determine the success of the biotinylation procedure. The remaining bacterial solutions were then centrifuged at 8000 rpm for 10 minutes and resuspended in PBS three times to remove unbound (+)-Biotin N-hydroxysuccinimide ester.

Sugar-binding assay

50 μL of biotinylated bacterial cells (OD of 1) was added to every well of a sugar functionalised surface before incubation at 37 °C for 30 minutes. Unbound solution is then removed from each well prior to washing three times with PBS. 100 μL ExtrAvidin®-FITC (in a 1 in 200 dilution in PBS) was then added to every well prior to incubation at 37 °C for 1 hour. Unbound solution was then removed and each well washed thoroughly three times with PBS before fluorescence readings were taken using a BioTek Synergy HT multi-detection microplate reader with excitation and emission wavelengths of 485 and 528 nm respectively.

Linear Discriminant Analysis

Every bacterial species/strain was added to every surface as described and each samples was repeated 8 times to generate a training matrix which was subjected to a classical linear discriminant analysis using the dapc function in the adegenet package

(version 1.4-2)[1] in the open source statistical package R (version 3.1.3).[2] All graphs were plotted in OriginPro.

Blind culture identification

A blind culture of one of the bacterial species was prepared and biotinylated as described. Biotinylated samples were then added to all the functionalised surfaces in triplicate as described. After fluorescence measurements were taken this data was classified using the linear discriminant analysis model produced by using the predict.dapc function in the adegenet package (version 1.4-2)[1] in the open source statistical package R (version 3.1.3).[2]

References

1. T. Jombart, C. Collins, P. Solymos, I. Ahmed, F. Calboli and A. Cori, *adegenet: an R package for the exploratory analysis of genetic and genomic data*, http://adegenet.r-forge.r-project.org/.
2. R. D. C. Team, *R: A Language and Environment for Statistical Computing. Vienna, Austria : the R Foundation for Statistical Computing*, http://www.R-project.org/.
